# Effects of Horsetail (*Equisetum arvense*) and Spirulina (*Spirulina platensis*) Dietary Supplementation on Laying Hens Productivity and Oxidative Status

**DOI:** 10.3390/ani11020335

**Published:** 2021-01-28

**Authors:** Vincenzo Tufarelli, Payam Baghban-Kanani, Saba Azimi-Youvalari, Babak Hosseintabar-Ghasemabad, Marina Slozhenkina, Ivan Gorlov, Alireza Seidavi, Tugay Ayaşan, Vito Laudadio

**Affiliations:** 1 Department of DETO, Section of Veterinary Science and Animal Production, University of Bari “Aldo Moro”, Valenzano, 70010 Bari, Italy; vito.laudadio@uniba.it; 2 Department of Animal Science, University of Tabriz, Tabriz 5166616471, Iran; p.baghbankanani@tabrizu.ac.ir (P.B.-K.); b.ht@tabrizu.ac.ir (B.H.-G.); 3 Department of Animal Science, Urmia University, Urmia 5756151818, Iran; s.azimiyouvalari@urmia.ac.ir; 4 Volga Region Research Institute of Manufacture and Processing of Meat-and-Milk Production, 400131 Volgograd, Russia; novyebio@mail.ru (M.S.); niimmp@mail.ru (I.G.); 5 Department of Animal Science, Rasht Branch, Islamic Azad University, Rasht 41335-3516, Iran; 6 Osmaniye Korkut Ata University, Kadirli Academy of Applied Sciences, 80000 Osmaniye, Turkey; tayasan@gmail.com

**Keywords:** laying hens, feed additives, egg quality, oxidative status

## Abstract

**Simple Summary:**

In the last decade, the interest in plants, plant extracts, and derived phytochemicals as dietary additives for poultry has significantly increased. This study aimed to investigate the effects of different dietary levels of both horsetail and spirulina on performance, egg quantitative and qualitative traits, serum and yolk cholesterol, and antioxidant status of laying hens. Based on the findings, supplementing laying hen diet with horsetail and spirulina did not have a detrimental effect on productive parameters.

**Abstract:**

The purpose of this study was to examine the effect of dietary horsetail (*Equisetum arvense*) and spirulina (*Spirulina platensis*) supplementation on performance, egg quality, serum biochemical and antioxidant status of laying hens. A total of 648, 63-week-old Hy-Line W-36 layers were divided into nine groups with eight replicates per group (nine birds per replicate). A feeding trial was conducted under completely randomized design with factorial arrangement 3 × 3 consisting of three different dietary levels of horsetail supplementations (0, 0.25, and 0.50%, respectively) in combination with three levels of spirulina (0, 1, and 2%, respectively). Results showed that feed intake, egg production, egg weight and mass, and feed conversion ratio were not significantly affected by the dietary treatments. Eggshell thickness, strength, and yolk color were significantly improved in diets supplemented with 0.5% horsetail and 2% spirulina and their interactions. Egg yolk cholesterol was not significantly different among groups; however, a significant reduction was found when fed 2% spirulina. Serum aspartate aminotransferase (AST) and alanine aminotransferase (ALT) concentration decreased by supplementing 2% spirulina in diet; also, spirulina increased total superoxide dismutase (TSOD) and total antioxidant capacity (TAC) in laying hens. Overall, the findings indicated that the combination of horsetail and spirulina could have potential for improving the egg’s physical parameters, whereas spirulina was more effective in improving blood traits and oxidative status.

## 1. Introduction

The interest in plants, plant extracts, and derived phytochemicals as dietary additives for poultry has significantly increased during the last decades [1]. Horsetail (*Equisetum arvense*) is a medicinal plant and is very popular in medicine worldwide as a beneficial plant for health disorders [2]. It is found in northern Iran under mild conditions [3]. Horsetail is usually used as a silica supplement (oxide form of silicon), which is about 25% of plant dry matter. Researchers assume that some of the benefits of horsetail are due to silica [4]. Silica improves the calcification process and bone and shell deposition [5]. Horsetail contains high amounts of polyphenols, fixed and volatile oils, and a variety of pharmacologically active substances [2,6].

Spirulina (*Spirulina platensis*) represents cyanobacterium cultivated for decades due to its valuable nutrient content [7]. Moreover, these algae are dietary supplements that have many positive effects on both humans and animals; thus, it could be an appropriate feed supplement in laying hen diets [8], but also an alternative protein and mineral source that enhances egg and meat quality without detrimental effects [9,10,11]. 

A previous study has demonstrated health effects of spirulina as a probiotic agent and immune system booster [12]. This marine alga is also supplemented to livestock species in order to improve growth traits, including the qualitative characteristics of poultry products [8].

To date, no applied studies on horsetail in layer diet have been conducted. Moreover, there is not much information on the effect of supplementing horsetail and spirulina together in combination in hens’ rations, except the independent effects of spirulina. Therefore, the present trial was conducted to investigate the effects of different dietary levels of both horsetail and spirulina on performance, egg quantitative and qualitative traits, blood and egg yolk cholesterol, and antioxidant status of laying hens. 

## 2. Materials and Methods

### 2.1. Animals and Diets

A total of 648, 63-week-old Hy-Line W-36 White Leghorn laying hens, having an average BW of 1550 ± 30 g at the beginning of the experiment, were randomly allocated to nine treatments (eight replicates with nine subjects) in a 3 × 3 factorial arrangement design. Hens were randomly assigned to cages so that there were eight replications. Each replicate consisted of three adjoining cages (30 × 40 × 42 cm) with three hens per individual cage for a total of nine hens/replicate. Each diet was formulated with UFFDA software [13]. The feeding trial was conducted under completely randomized design with factorial arrangement 3 × 3 consisting of three different dietary levels of horsetail supplementations (0, 0.25, and 0.50%, respectively) in combination with three levels of spirulina (0, 1, and 2%, respectively). The ingredients and chemical composition of basal-diets are reported in Table 1. These basal-diets were not fed to laying hens, but were used to formulate the different feed mixtures containing the above-mentioned levels of horsetail and spirulina and their combination. Feed and water were offered ad libitum. The horsetail powder was provided from a commercial company (Darvash Giah Khazar, Rasht, Iran), and the chemical composition of horsetail is presented in Table 2. The algae used in the study was *Spirulina platensis*, pure *Spirulina platensis* powder (spirulina ~100%) purchased from the Nour Darou Company, Gonbad-Kavous, Golestan, Iran. The composition of *Spirulina platensis* powder is reported in Table 3. The trial lasted 12 weeks.

### 2.2. Sample Collection

During the trial, average feed intake, egg weight, mass and production, feed conversion ratio (FCR), and mortality were assessed. The hen BW was determined at beginning and end of study. Eggs from individual layers were collected daily and weighed [14]. Before any egg quality parameters were taken in lab facilities, eggs were stored at room temperature. Egg quality parameters were assessed 24 h after collection, and included eggshell strength, shell thickness, yolk index and color, and Haugh unit [14]. 

A total of 48 eggs per treatment were taken randomly for egg yolk cholesterol analysis as previously described [14]. At the end of the study, blood was sampled to determine antioxidant capacity, malondialdehyde (MDA) concentrations, cholesterol, triglyceride, aspartate aminotransferase (AST), and alanine aminotransferase (ALT) as described by Baghban-Kanani et al. [14].

### 2.3. Antioxidant Parameters

Antioxidant capacity TAC, TSOD, and glutathione peroxidase (GSH-Px) was assessed in blood using kits (Randox, Germany) and according to Winterbourn et al. [15]. Serum lipid peroxidation was analyzed as reported by Kei [16] and Yagi [17], but using 1,1,3,3-tetraethoxypropane as standard. Serum lipid peroxidation values are expressed in MDA as nmol/mL plasma.

### 2.4. Statistical Analysis 

Data were analyzed using the one-way procedure of ANOVA with 9 treatments and 8 replicates having 9 hens per replicate, using the GLM procedure of SAS (SAS 9.3; 2001; SAS Institute Inc., Cary, NC, USA) [18] for Windows. Means were compared by the Duncan’s multiple range tests with *p* < 0.05.

## 3. Results and Discussion

### 3.1. Performance Traits 

The dietary effect on performance and egg parameters recorded for the 70-day feeding period is shown in Table 4. There was no effect on horsetail nor spirulina levels (*p* > 0.05) on feed consumption, egg production, weight, mass, and FCR during the trial period. The significant effects on performance and egg parameters due to horsetail × spirulina interactions were also not observed. Horsetail and spirulina did not influence laying hens’ productive parameters. These data are in agreement with findings of Garcia et al. [19] and Zahroojian et al. [20] who indicated that pigment inclusion was not correlated to productive traits. Conversely to our findings, Selim et al. [9] reported that spirulina enhanced egg production and quality. These contrast results are presumably related to differences in strains and age of laying hens. To the best knowledge, no comparable data were found in the available studies on the influence of horsetail on performance and egg parameters in laying hens.

### 3.2. Egg Quality Parameters

The egg qualitative measurements are summarized in Table 5. There were no differences among groups on egg qualitative parameters investigated (*p* > 0.05). Conversely, significant differences of shell thickness, strength, and yolk color were observed in hens fed different dietary horsetail and spirulina levels. The interactions between horsetail × spirulina on eggshell thickness and strength and yolk color were also significant (*p* < 0.05). Hens fed diets containing 0.50% horsetail and 2% spirulina recorded the highest shell thickness, shell strength, and yolk color (*p* < 0.05). For worldwide egg producers, having good eggshell quality is a critical point related to their economic viability [21]. There is no available report using horsetail in laying hens’ diet, thus our study may be considered in new investigation. Silica accelerates the process of calcification and deposition in bone and shell, improving bone matrix quality, and facilitating bone mineralization [5]. Therefore, since horsetail is well known to have high silica amounts in plants [4], its application in cage layer fatigue treatment is recommended.

Additionally, as shown in Table 2, horsetail is a good source of calcium, protein, fiber, and an array of other minerals which improve bone matrix quality and mineralization. Conversely, from our trial it was assessed that feeding of 2% spirulina enhanced the eggshell thickness and strength. These results are in agreement with Selim et al. [9] who showed that supplementing *Spirulina platensis* powder at 0.3% in diet had a beneficial effect on eggshell thickness. This may be due to the effects of spirulina’s high calcium content. With this in mind, Williamson and Burkitt [22] explained that the calcium content of spirulina is ~26 times more potent than milk. In contrast to the present study, Zahroojian et al. [20] reported that *Spirulina platensis* had no significant effect on egg traits when laying hens were fed up to 2.5% in diet. Moreover, a study on the effect of spirulina on eggshell strength conducted by Dogan et al. [23] revealed the addition up to 2% did not have an effect on the physical traits of Japanese quail eggs. Egg yolk color is important for consumer satisfaction, and consumers around the world usually prefer yolk color ranging from golden yellow to orange [24,25]. The improvement of yolk color when fed horsetail can be explained by the higher content of flavonoids in diets, thus in horsetail. It was demonstrated by Garcia et al. [19] that feeding poultry diets that included flavonoids corresponded to higher pigment deposition in yolk, which determined the color intensity. In this regard, the effects of spirulina on yolk color are mainly due to the content of carotenoid pigments, mostly beta-carotene. Our results agreed with those of Selim et al. [9] and Zahroojian et al. [20] indicating that carotenoids are capably deposited in yolk. Similarly, Ross and Dominy [26] observed that egg yolk color escalates linearly with increased dietary spirulina levels. 

### 3.3. Egg-Yolk Cholesterol

The effects of experimental diets on yolk weight and cholesterol are presented in Table 6. It was revealed that diets had no effect (*p* > 0.05) on yolk weight, but the lowest egg yolk cholesterol values were recorded when diets were supplemented with 2% spirulina (*p* < 0.05). Spirulina or its extracts have cholesterol reducing effects in mice, rats, and also in layers [27]. Moreover, this marine alga has been found to be a way to modulate egg quality in order to meet consumer preferences. For example, total egg cholesterol may be reduced by dietary spirulina, and this was mostly due to high content of n-3 polyunsaturated fatty acids [28]. In a study, Dogan et al. [23] reported in laying Japanese quails that egg yolk cholesterol dropped significantly when fed up to 2.0% of spirulina. In contrast, Zahroojian et al. [20] reported no significant reductions in egg yolk cholesterol as the level of dietary spirulina increased. 

### 3.4. Blood Biochemical Parameters

Table 7 shows the influence of diet on blood parameters in hens. In this study, serum triglyceride and cholesterol levels were not influenced by treatments (*p* > 0.05). The interactions between horsetail × spirulina on serum AST, ALT, triglyceride, and cholesterol was not significant when compared to control-diet (*p* > 0.05). The spirulina inclusion level of 2% decreased AST and ALT concentrations in serum (*p* < 0.05). The assessed low serum ALT and AST levels in hens fed spirulina demonstrated the hepatoprotective action of these marine algae due to antioxidant property. The liver is the main metabolic organ of the body, and more severe liver damage releases higher amounts of liver enzymes. The decreased level of serum enzyme such as AST and ALT indicates lower damage of hepatocytes [29].

### 3.5. Antioxidant Status

The influence of diets on serum GSH-Px activity, TSOD, MDA, and TAC is reported in Table 8. Significant differences were found adding 2% spirulina to diet on TSOD and TAC. The concentrations of TSOD and TAC increased with increasing level of spirulina (*p* < 0.05), whereas there were differences in GSH-Px activity, MDA, TSOD, and TAC due to horsetail × spirulina interactions (*p* > 0.05). There is great interest in safe and new natural antioxidants as feed additives to reduce oxidative deterioration of foodstuffs and to cell damage [30,31]. Moreover, natural feed additives may support poultry in resisting to environmental stressors and especially in supporting poultry productions, such as lowering egg yolk cholesterol, and also improving the antioxidant status of livestock species [32]. Increased activity of TSOD and TAC enzymes may also support the immune system of laying hens. According to available reports indicating a direct influence of spirulina in poultry diet [33], this study suggests that spirulina has great antioxidant potential and is capable of determining the positive effects on the health of laying hens.

## 4. Conclusions

According to the present findings, supplementing laying hen diet with horsetail and spirulina did not have detrimental effects on productive parameters. Feeding of horsetail and spirulina at the level of 0.50% and 2%, respectively, was effective in increasing egg physical traits (eggshell thickness and strength) and yolk color. Moreover, adding 2% spirulina to the hen diet could be suggested given lowering effect on yolk total cholesterol, a positive aspect also related to consumer health. Additionally, spirulina decreased in blood AST and ALT and increased TSOD activity, as well as TAC amount. However, further studies should be conducted on this subject to deeply specify the effective dose of horsetail in laying hens, as well as in other poultry species. 

## Figures and Tables

**Table 1:** The ingredients and nutritional composition of basal-diets.

Ingredients, %	Control	Horsetail	Spirulina
Yellow corn	48.90	35.21	52.51
Wheat	20.00	20.00	20.00
Soybean meal (48% crude protein)	18.73	16.50	-
Horsetail	-	15.00	-
Spirulina	-	-	15.00
Calcium carbonate	10.00	9.40	9.40
Dicalcium phosphate	1.52	1.52	1.52
Salt	0.32	0.32	0.32
Soybean oil	1.06	1.30	0.50
Vitamin–mineral mix	0.50	0.50	0.50
DL-Met	0.12	0.05	0.05
L-Lys HCl	0.05	0.20	0.20
Chemical composition (%)			
AME_n_ (kcal/kg)	2763	2698	2875
Crude protein	15.49	15.60	15.62
Crude fiber	2.60	3.95	2.23
Ca	4.36	4.33	4.41
Available P	0.35	0.35	0.36
Na	0.16	0.15	0.16
Available Met	0.34	0.25	0.35
Available Met+Cys	0.63	0.52	0.94
Available Lys	0.75	0.79	0.73

Control: basal-diet without feed supplements; Horsetail: basal-diet including 15% horsetail, not fed to animals but used to formulate the different feed mixtures (0, 0.25, and 0.50%); Spirulina: basal-diet including 15% spirulina, not fed to animals but used to formulate the different feed mixtures (0, 1, and 2%); AME_n_: nitrogen-corrected apparent metabolizable energy.

**Table 2:** Nutrient ^1^ and phytochemical ^2^ content of horsetail.

Specification	
Dry matter (%)	92.14
Crude protein (%)	13.53
Crude fat (%)	1.56
Crude fiber (%)	11.90
Ash (%)	18.34
Calcium (%)	1.32
Phosphorus (%)	0.20
Magnesium	0.97
Sodium (%)	0.72
Starch (%)	11.71
Sugar (%)	3.54
Carbohydrates (%)	46.81
Selenium (mg/100 g)	1.00
Iron (mg/100 g)	29.40
Copper (mg/100 g)	4.30
Manganese (mg/100 g)	1.50
Zinc (mg/100 g)	7.50
Total Polyphenols (%)	1.46
Tannins (%)	0.26
Vitamin E (μg/100 g)	70.00
Linalool (%)	0.33
Benzyl acetate (%)	3.65
Cyclamen aldehyde (%)	2.51
Dimethylbenzylcarbinyl butyrate (%)	11.51
2-Nonenal, 2-pentyl (%)	0.19
Undecalactone (%)	81.80

^1^ Analysis of all nutrient values by ViroMed Central Analytical Lab (Samaneh Payesh Salamat Lab), Pardis Technology Park, Tehran, Iran. ^2^ Phytochemical analysis by KSP Lab (Kimia Shengerf Pars Lab), Tehran, Iran.

**Table 3:** Spirulina nutrient content.

Chemical Composition (% on Dry Matter)	
Moisture	7.22
Protein	57.61
Fat	5.31
Fiber	3.80
Ash	13.8
Mineral concentrations (%)	
Calcium	2.20
Phosphorus	0.40
Magnesium	0.85
Iron	1.23
Manganese	0.80
Zinc	1.87
Vitamins and carotenoids (μg/100 g)	
Total carotenoids	680
Vitamin A	134
Vitamin E	105
Ascorbic acid	824

Data were obtained from the manufacturer (The Nour Darou Company, Gonbad-Kavous, Golestan, Iran).

**Table 4:** Influence of treatments on feed intake and efficiency, and egg production.

Item	Feed Intake (g/d)	Egg Production (%)	Egg Weight (g)	Egg Mass (g/d)	FCR
Horsetail %					
0	108.65	69.42	60.22	41.81	2.59
0.25	108.84	69.29	60.30	41.78	2.60
0.50	109.33	70.14	60.55	42.47	2.57
SEM	0.46	0.26	0.16	0.18	0.01
Spirulina %					
0	108.36	69.36	60.07	41.66	2.60
1	109.17	69.61	60.34	42.00	2.59
2	109.30	69.89	60.66	42.39	2.57
SEM	0.46	0.26	0.16	0.18	0.01
Horsetail × Spirulina					
0 × 0	107.90	68.96	59.99	41.37	2.60
0 × 1	108.80	69.37	60.17	41.74	2.60
0 × 2	109.24	69.94	60.49	42.31	2.58
0.25 × 0	108.36	69.36	60.11	41.69	2.59
0.25 × 1	109.27	69.24	60.28	41.74	2.61
0.25 × 2	108.90	69.27	60.51	41.92	2.59
0.50 × 0	108.80	69.75	60.11	41.92	2.59
0.50 × 1	109.44	70.22	60.56	42.53	2.57
0.50 × 2	109.75	70.45	60.98	42.96	2.55
SEM	0.79	0.46	0.28	0.31	0.02
*p*-value					
Horsetail	0.55	0.09	0.32	0.09	0.43
Spirulina	0.30	0.38	0.17	0.12	0.58
H × S	0.85	0.33	0.30	0.19	0.90

Means within a row with no superscripts do not differ (*p* > 0.05); FCR: kg feed/kg egg; H × S: Horsetail × Spirulina interaction.

**Table 5:** Influence of diet on egg qualitative traits.

Item	Shell Thickness (mm)	Eggshell Strength (kg/cm^2^)	Shape Index (%)	Haugh Unit	Yolk Color Score
Horsetail %					
0	0.29 ^b^	2.54 ^b^	61.06	78.47	8.38 ^b^
0.25	0.29 ^b^	2.59 ^b^	61.24	78.61	8.58 ^a,b^
0.50	0.30 ^a^	2.78 ^a^	62.27	78.92	9.33 ^a^
SEM	0.002	0.02	0.22	0.44	0.35
Spirulina %					
0	0.29 ^b^	2.59 ^b^	61.01	78.20	7.37 ^b^
1	0.29 ^b^	2.63 ^a,b^	61.24	78.83	8.75 ^a^
2	0.30 ^a^	2.69 ^a^	61.32	78.97	9.87 ^a^
SEM	0.002	0.02	0.22	0.44	0.35
Horsetail × Spirulina					
0 × 0	0.28 ^b^	2.50 ^c^	61.00	78.10	6.62 ^b^
0 × 1	0.29 ^ab^	2.54 ^c^	61.08	78.89	8.87 ^a,b^
0 × 2	0.30 ^a, b^	2.59 ^b,c^	61.10	78.41	10.25 ^a^
0.25 × 0	0.29 ^a, b^	2.57 ^b,c^	61.04	78.37	7.00 ^a,b^
0.25 × 1	0.29 ^a, b^	2.60 ^b,c^	61.37	78.55	8.25 ^a,b^
0.25 × 2	0.29 ^a, b^	2.61 ^b,c^	61.41	78.90	9.00 ^a,b^
0.50 × 0	0.30 ^a, b^	2.71 ^a,b^	61.01	78.12	8.50 ^a,b^
0.50 × 1	0.30 ^a, b^	2.76 ^a,b^	61.26	79.04	9.12 ^a,b^
0.50 × 2	0.31 ^a^	2.86 ^a^	61.45	79.61	10.37 ^a^
SEM	0.003	0.04	0.39	0.76	0.61
*p*-value					
Horsetail	0.004	0.001	0.770	0.760	0.050
Spirulina	0.030	0.050	0.610	0.420	0.001
H × S	0.001	0.001	0.980	0.910	0.004

^a–c^ Means within a row with no superscripts do not differ (*p* > 0.05). H × S: Horsetail × Spirulina interaction.

**Table 6:** Influence of treatments on egg yolk cholesterol and weight.

Item	Yolk Weight (g)	Yolk Cholesterol (mg/yolk)	Yolk Cholesterol (mg/g yolk)
Horsetail %			
0	17.82	220.50	12.37
0.25	17.54	220.00	12.46
0.50	17.82	218.58	12.33
SEM	0.14	5.28	0.27
Spirulina %			
0	17.70	228.10 ^a^	12.87 ^a^
1	17.73	222.60 ^a,b^	12.54 ^a,b^
2	17.75	208.38 ^b^	11.75 ^b^
SEM	0.14	5.28	0.27
Horsetail × Spirulina			
0 × 0	17.74	230.57	13.00
0 × 1	17.86	218.98	12.25
0 × 2	17.86	211.97	11.87
0.25 × 0	17.62	224.64	12.74
0.25 × 1	17.50	221.04	12.62
0.25 × 2	17.50	210.07	12.00
0.50 × 0	17.75	229.11	12.87
0.50 × 1	17.84	227.79	12.75
0.50 × 2	17.88	203.11	11.37
SEM	0.24	9.14	0.47
*p*-value			
Horsetail	0.26	0.95	0.95
Spirulina	0.97	0.02	0.01
H × S	0.91	0.38	0.26

^a,b^ Means within a row with no superscripts do not differ (*p* > 0.05). H × S = Horsetail × Spirulina interaction.

**Table 7:** Influence of diets on blood parameters.

Item	AST (U/l)	ALT (U/l)	Triglycerides (mg/dL)	Cholesterol (mg/dL)
Horsetail %				
0	214.52	4.58	97.12	99.43
0.25	213.48	4.56	95.42	98.15
0.50	213.50	4.77	97.03	100.48
SEM	0.41	0.13	1.37	1.71
Spirulina %				
0	215.10 ^a^	4.90 ^a^	98.19	102.27
1	213.73 ^a,b^	4.61 ^a,b^	96.14	98.40
2	212.68 ^b^	4.40 ^b^	95.24	97.39
SEM	0.41	0.13	1.37	1.71
Horsetail × Spirulina				
0 × 0	215.97	5.08	99.06	103.96
0 × 1	213.61	4.51	96.89	97.55
0 × 2	213.97	4.15	95.41	96.79
0.25 × 0	213.36	4.55	96.46	98.88
0.25 × 1	213.97	4.69	95.26	98.47
0.25 × 2	213.09	4.45	94.54	97.09
0.50 × 0	215.97	5.08	99.06	103.95
0.50 × 1	213.65	4.63	96.26	99.17
0.50 × 2	213.97	4.61	95.78	98.29
SEM	0.71	0.23	2.38	2.97
*p*-value				
Horsetail	0.13	0.48	0.62	0.63
Spirulina	0.03	0.04	0.30	0.11
H × S	0.08	0.17	0.88	0.57

^a,b^ Means within a row with no superscripts do not differ (*p* > 0.05). H × S = Horsetail × Spirulina interaction; AST: Serum aspartate aminotransferase; ALT: alanine aminotransferase.

**Table 8:** Effect of experimental diets on serum glutathione peroxidase (GSH-Px) activity, malondialdehyde (MDA), total superoxide dismutase (TSOD), and total antioxidant capacity (TAC).

Items	GSH-Px (U/mL)	MDA (nmol/mL)	TSOD (U/mL)	TAC (U/mL)
Horsetail %				
0	822.14	6.23	163.07	7.88
0.25	822.46	6.42	163.57	7.80
0.50	823.45	6.31	163.38	7.76
SEM	1.67	0.12	1.29	0.04
Spirulina %				
0	821.25	6.53	160.98 ^b^	7.73 ^b^
1	822.81	6.30	163.57 ^a,b^	7.79 ^a,b^
2	824.00	6.12	165.47 ^a^	7.92 ^a^
SEM	1.67	0.12	1.29	0.04
Horsetail × Spirulina				
0 × 0	819.09	6.54	161.09	7.75
0 × 1	822.09	6.22	162.71	7.88
0 × 2	825.25	5.92	165.42	8.02
0.25 × 0	822.26	6.53	160.60	7.72
0.25 × 1	823.14	6.39	164.42	7.76
0.25 × 2	822.02	6.33	165.69	7.80
0.50 × 0	822.43	6.53	161.25	7.73
0.50 × 1	823.19	6.29	163.59	7.74
0.50 × 2	824.74	6.12	165.31	7.94
SEM	2.89	0.22	2.25	0.08
*p*-value				
Horsetail	0.84	0.57	0.96	0.19
Spirulina	0.50	0.08	0.05	0.02
H × S	0.92	0.52	0.60	0.13

^a,b^ Means within a row with the same or no superscripts do not differ significantly (*p* > 0.05). H × S = Horsetail × Spirulina interaction.

## Data Availability

Data presented in this study are available on fair request from the respective author.
